# Revisiting the oxygen reactivity index in traumatic brain injury: the complementary value of combined focal and global autoregulation monitoring

**DOI:** 10.1186/s13054-025-05261-6

**Published:** 2025-01-12

**Authors:** Teodor Svedung Wettervik, Erta Beqiri, Anders Hånell, Stefan Yu Bögli, Ihsane Olakorede, Xuhang Chen, Adel Helmy, Andrea Lavinio, Peter J. Hutchinson, Peter Smielewski

**Affiliations:** 1 Department of Medical Sciences, Section of Neurosurgery, Uppsala University, 751 85 Uppsala, Sweden; 2 Brain Physics Laboratory, Department of Clinical Neurosciences, Division of Neurosurgery, University of Cambridge, Cambridge, UK; 3 Department of Clinical Neurosciences, Division of Neurosurgery, Addenbrooke’s Hospital, University of Cambridge, Cambridge, UK; 4 Neurosciences and Trauma Critical Care Unit, Addenbrooke’s Hospital, Cambridge University Hospitals, Cambridge, UK

**Keywords:** Brain tissue oxygenation, Oxygen reactivity index, Pressure reactivity index, Traumatic brain injury

## Abstract

**Background:**

The oxygen reactivity index (ORx) reflects the correlation between focal brain tissue oxygen (pbtO_2_) and the cerebral perfusion pressure (CPP). Previous, small cohort studies were conflicting on whether ORx conveys cerebral autoregulatory information and if it is related to outcome in traumatic brain injury (TBI). Thus, we aimed to investigate these issues in a larger TBI cohort.

**Methods:**

425 TBI patients with intracranial pressure (ICP)- and pbtO_2_-monitoring for at least 12 h, who had been treated at Addenbrooke’s Hospital, Cambridge, UK, were included. Association between ORx and ICP, pressure reactivity index (PRx), CPP, ΔCPPopt (actual CPP-CPPopt [PRx based optimal CPP]), and pbtO_2_ were evaluated with generalized additive models (GAMs). Association between ORx and outcome (Glasgow Outcome Scale [GOS]) was investigated with logistic regressions and heatmaps for those 239 patients with GOS data.

**Results:**

GAMs showed that ORx increased with higher ICP, PRx above + 0.30, CPP below 60–70 mmHg, and negative ΔCPPopt. In contrast to PRx, ORx did not increase at higher CPP. In outcome heatmaps, there was a transition towards unfavourable outcome when ORx exceeded + 0.50, particularly for longer durations, and in combination with high ICP, high PRx, low CPP, negative ΔCPPopt, and low pbtO_2_. In multivariable logistic regressions, higher ORx was associated with increased mortality.

**Conclusions:**

ORx seemed to be sensitive to the lower, but not the upper, limit of autoregulation, in contrast to PRx which was sensitive to both. The combination of high values for both ORx and PRx was particularly associated with worse outcome and, thus, ORx may provide a complementary value to the global index PRx. ORx could also be useful to determine the safe and dangerous perfusion target intervals.

**Supplementary Information:**

The online version contains supplementary material available at 10.1186/s13054-025-05261-6.

## Introduction

Patients with moderate-to-severe traumatic brain injury (TBI) often develop cerebral autoregulatory (CA) impairment, which makes them vulnerable to ischaemia and hyperaemia [1–3]. The CA capacity may differ between patients and change over time [4, 5]. Therefore, it is challenging to set the cerebral perfusion pressure (CPP) target for these patients [1, 6, 7]. Currently, the pressure reactivity index (PRx), i.e., the moving correlation coefficient between slow changes in arterial blood pressure (ABP) and intracranial pressure (ICP), is the most well-known and validated CA metric in the neurocritical care (NCC) setting [5, 8–11]. High PRx indicates impaired cerebrovascular reactivity and has consistently been associated with poor outcome [5, 10]. Furthermore, PRx changes with CPP in a U-shaped way and the CPP associated with the lowest PRx is suggested to be optimal (CPPopt) [2, 12, 13].

In addition to PRx, other continuous monitoring methods for CA have been suggested in TBI [1–3]. In particular, the oxygen reactivity index (ORx) is the moving Pearson correlation coefficient originally based on 30 s-averages of CPP and focal brain tissue oxygen (pbtO_2_) over the last hour [14–16]. In case of CA impairment, CPP and pbtO_2_ are expected to change in the same direction, resulting in a positive ORx. However, ORx is expected to remain close to zero when CA is intact, since changes in CPP should not alter pbtO_2_ as both cerebral blood flow (CBF) and diffusive oxygen delivery are expected to remain stable. The physiological meaning of negative ORx remains elusive. Initial studies indicated that elevated ORx was both associated with higher PRx and worse outcome [14], but others have not been able to replicate these findings [16–18]. The validity of ORx as a metric of CA has also been questioned, since it does not appear to reflect ABP slow waves [19]. Furthermore, ORx is based on focal measurements of pbtO_2_, which reflect tissue oxygen levels in a very small area in the brain. This region may not be representative of the global brain state, taking into account that TBI is associated with significant spatial variation in injury patterns and physiological disturbances [20].

Since there are only a handful of small cohort studies on this topic in TBI [14, 16–18], we argue that no firm conclusions can be made regarding its potential and limitations. At the same time, in other acute brain injuries, such as aneurysmal subarachnoid haemorrhage (aSAH), ORx has turned out to be a strong predictor of clinical outcomes such as the risk of developing delayed cerebral ischaemia or worse functional recovery [15, 21]. Thus, we believe that there is a need to clarify the role of ORx in TBI, using a large patient cohort that allows for more accurate and in-depth analyses. Therefore, the primary aim of this TBI study was to investigate how ORx varies with other cerebral physiological variables including PRx. The secondary aim was to investigate if ORx is related to outcome and if this depends on concurrent disturbances in other cerebral physiological variables. We hypothesized that ORx would be particularly sensitive to ischaemia, characterized by high ICP, low CPP, and impaired autoregulation, but not to hyperaemia, since pbtO_2_ is expected to become saturated at high CPP and CBF [20, 22]. We also hypothesized that higher ORx would be associated with worse outcome, particularly in combination with high ICP, low CPP, high PRx, CPP below CPPopt, and low pbtO_2_.

## Materials and methods

### Patients and study design

In this observational study, adult TBI patients with at least 12 h of ICP and pbtO_2_ monitoring data, who had been treated in the NCC unit at Addenbrooke’s Hospital, Cambridge, UK, between 2002 and 2022 were screened in the Brain Physics research database. We retrieved ABP, ICP and pbtO_2_ monitoring data and the following clinical descriptors: age, Glasgow Coma Scale (GCS), sex, pupillary reactivity, surgery with decompressive craniectomy, and Glasgow Outcome Scale (GOS). The study follows two lines of investigations. All eligible patients were included in the analyses on ORx in relation to other cerebral physiological variables, while only those with GOS data were considered in the analyses of the association between ORx and outcome (Additional File 1). Thus, of 461 eligible patients with ICP and pbtO_2_ data, those 425 with more than 12 h of ICP/pbtO_2_ monitoring time were included in the physiological analyses of ORx, while a subset of 239 patients who had GOS data were included in the analyses of ORx and outcome.

### Management protocol

The management protocol has been described in detail in previous studies [20, 23]. The protocol underwent some changes throughout the 20-year period [23], including the alteration in reference point of ABP (from heart to tragus level) and general improvements in healthcare. ICP- and pbtO_2_-monitoring were considered in unconscious TBI patients. Overall, CPP was targeted above 60 mmHg, ICP below 20 or 25 (for last-tier treatments; barbiturates and decompressive craniectomy) mmHg, pbtO_2_ above 15–20 mmHg, partial pressure of carbon dioxide within 4.5–5 kPa, and arterial glucose within 6–8 mmol/L. The pbtO_2_ target was mainly managed by ICP-lowering and CPP-augmenting measures. PRx and CPPopt, available at the bedside since 1999 and 2012, respectively, were not actively targeted, but could be taken into account based on the discretion of the treating physician. ORx was not available at the bedside.

### Outcome

Clinical outcome was evaluated using GOS assessments six months after the TBI, either by clinical follow-up or telephone interviews. GOS ranges from 1 (deceased) to 5 (good recovery) [24, 25] and was dichotomized as survival vs. mortality (GOS 2 to 5 vs. 1) and favourable vs. unfavourable outcome (GOS 4 to 5 vs. 1 to 3).

### Collection and processing of cerebral physiological data

ICP (Codman ICP MicroSensor, Codman & Shurtleff, Raynham, Massachusetts) and pbtO_2_ (Licox, Integra Neurosciences, Andover, UK) were monitored with intraparenchymal probes mainly in the right frontal lobe, while avoiding insertion into lesions, using the Cambridge Cranial Access Device. ICP was also occasionally monitored with an external ventricular drain. ABP was monitored in the radial or femoral artery (Baxter Healthcare, Deerfield, Illinois) at heart (2002–2015) or tragus (2015–2022) level, respectively. Physiological data were streamed in real time at 50–240 Hz from the monitors into the laptop with ICM + software (ICM + software, Cambridge Enterprises, University of Cambridge, UK) (https://icmplus.neurosurg.cam.ac.uk), which provided data integration, storage, and de-identification for the Brain Physics Lab research database. The physiological data were curated manually and automatically as previously described [20] and subsequently down-sampled to 0.1 Hz by coarse graining using 10 s, non-overlapping averages. The good monitoring time (GMT) was defined as the remaining time after exclusion of these artefacts or any other gaps in the data. PRx was calculated as the moving Pearson correlation coefficient of 30 consecutive 10-s average values of ABP and ICP, and updated every minute [8, 10]. CPPopt was calculated according to the multi-window weighted algorithm based on a data buffer of 2–8 h and defined as the corresponding CPP with the lowest PRx [26]. ∆CPPopt was defined as the difference between actual CPP and CPPopt. ORx was calculated as the moving Pearson correlation coefficient of 120 consecutive 30 s-values of pbtO_2_ and CPP over the last hour, and updated every minute [14–16]. However, since 2018, the updated Licox monitor only sent 60 s-values. In these cases, up-sampling to 30 s pbtO_2_-values (using the same pbtO_2_ value for both 30 s-time points within each 60 s-interval) were done before the ORx calculations. The final, resulting, minute-by-minute and hour-by-hour mean values of all the parameters were subsequently used for statistical analyses. The hourly values were also classified into blocks for periods of continuous monitoring without interruptions/data gaps. For the hourly data, separate variables of negative/positive ∆CPPopt (below/above 0) were defined. For example, when calculating hourly values of negative ∆CPPopt, every minute-value of ∆CPPopt > 0 was counted as 0 and every ∆CPPopt < 0 was counted as a positive mmHg (e.g., CPP at 80 mmHg and CPPopt at 90 mmHg yielded a positive value at 10 mmHg). Lastly, the mean values of ICP, PRx, ORx, CPP, CPPopt, and pbtO_2_ were calculated over the GMT.

### Visualization of outcome

The association between ORx and outcome was visualized using three different methods that have been developed in R-scripts [9, 11, 27, 28], as described below.

In the first approach [11], a single-variable outcome heatmap was created based on the %GMT of ORx, divided into 20 separate grid cells (range − 1 to + 1, 0.1 per grid cell). Thus, we explored the full ORx range for completeness, even if the physiological meaning of negative ORx may be less clear. The %GMT for each grid cell was correlated (Spearman) with patient outcome (GOS) [11]. To produce smoother images, each grid cell was divided into 3 * 3 sub cells, followed by application of a Gaussian kernel filter (standard deviation of 2 grid cells). The final values for each grid cell were translated into the jet colour range with red colour indicating a negative association with GOS and vice versa for blue colour. Due to the moderate correlation strength (r within ± 0.3), the jet colour scale was limited to this range. Grid cells were coloured as white if they had less than 5 patients with at least 5 min of GMT. A corresponding density heatmap was created by counting the number of observations within each grid cell, and then divided by the highest count among all grid cells, for each patient. A similar smoothing process was applied and the density heatmap was then coded using the jet colour scale (blue = frequent and red = rare).

In the second approach [9, 27, 29], the combined insult intensity and duration of ORx was analysed in relation to GOS. This method was based on a similar approach as was used by Guiza et al. [30], which had been modified by the Uppsala group [9, 27]. The heatmap was defined in terms of ORx ‘insult’ intensity (range − 0.5 to + 1.0, 0.1 per grid cell) and duration (range 0–120 min, 2 min per grid cell). The lowest intensity insult threshold of ORx was set at -0.5, since lower values were uncommon and episodes above such low threshold values for longer durations would be unspecific and could encompass almost anything. The upper insult threshold limit was set to explore the full positive ORx range. The insult duration threshold upper limit was set at 120 min, which was estimated to be a considerable time even for low-intensity insults to induce secondary brain injury. The number of insults per grid cell, e.g., ORx above + 0.50 for exactly 30 min, was counted for every patient and correlated with outcome. Negative correlation coefficients indicated an association with worse outcome and vice versa. A similar smoothing process, as above, was applied and the final correlation values were visualized using the jet colour scale (blue = favourable and red = unfavourable). Grid cells with less than 20 patients with at least one insult were coloured as white. A similar density heatmap was created as for the previous method, however, in this case, short insults were much more prevalent than longer insults. Therefore the logarithmic density was found to be more informative and was used instead [29].

In the third approach [27–29], combined insults of ORx together with ICP, PRx, CPP, ΔCPPopt, or pbtO_2_ were analysed in relation to outcome over the entire GMT. For the ORx/ICP plot, the grid was composed of 500 (20*25) cells based on ORx (range − 1 to + 1, 0.1 per grid cell) and ICP (range 0–50 mmHg, 2 mmHg per grid cell). The %GMT was calculated for all patients for all grid cells and was then correlated with GOS. Similar smoothing procedures were performed, as mentioned above, and the map was then coded according to the jet colour scale (blue = higher %GMT was favourable and red = higher %GMT was unfavourable). Due to the moderate correlation strength (r within ± 0.3), the jet colour scale was limited to this range. Grid cells were coloured white if they had less than 5 patients with at least 5 min GMT. In the combined insult plots, ORx was always divided into 20 grid cells of 0.1-intervals. This yielded heatmaps with 400 cells (20*20) for PRx (range − 1 to + 1, 0.1 per grid cell), 500 (20*25) grid cells for CPP (range 20 to 120, 4 mmHg per grid cell), 500 (20*25) grid cells for ∆CPPopt (range − 50 to + 50 mmHg, 4 mmHg per grid cell), and 300 (20*15) grid cells for pbtO_2_ (range 5–50 mmHg, 3 mmHg per grid cell). Density heatmaps were created in a similar way as for the first method.

### Statistical analysis

The statistical analyses were conducted in RStudio software (version 4.4.1) [31]. Descriptive data were presented as numbers (proportions) or means (± standard deviation [SD]). Potential differences in demography, injury severity, cerebral physiology, and treatments between the cohort with available vs. missing outcome data were compared with t- and chi-square tests, depending on the type of data. The association between ORx in relation to ICP, PRx, CPP, ∆CPPopt, and pbtO_2_ was analysed with generalized additive models (GAMs) with cubic spline, based on the restricted maximum likelihood, using minute-by-minute data (geom_smooth component of the R ggplot2 package). This approach allowed us to explore large amounts of data at a population level and to investigate potential non-linear associations between the variables. A GAM of the association between PRx and CPP was added to investigate if this differed compared to the GAM of ORx and CPP.

Linear mixed effect models (R package lme4) of hour-by-hour data with ORx as the dependent variable and a selection of the cerebral physiological variables as the independent variables were also explored. This approach allowed us to adjust for potential patient-related and temporal effects. Two separate models were used. One was based on absolute CPP values and included ICP, PRx (above/below + 0.30, i.e., approximately within the limit of CA [32]), CPP, and the interaction between ICP and CPP with PRx, as fixed effects. Patient ID and the specific monitoring sequence were used as random intercepts, while time from start of monitoring modelled the random slopes. Stepwise backward model reduction was pursued to exclude variables that were not significant (*p* > 0.05 in the ANOVA table). A similar model was done with negative/positive ∆CPPopt instead of CPP as a fixed effect. Conditional and marginal R^2^ (R package performance v 0.1.0.0) of these models were assessed [33].

Multivariable logistic regressions of favourable outcome and mortality with corresponding odds ratios (OR) and 95% confidence intervals (CI) were performed with age, GCS, PRx, and ORx as the independent variables. The former three variables were used to adjust for demography (age), injury severity (GCS), and global CA status (PRx). A *p* value below 0.05 was considered statistically significant.

## Results

### Demography, admission variables, treatments, and outcome

425 TBI patients were included in the physiological explorations, while only 239 of these had available outcome data. In the entire cohort of 425 TBI patients (Table 1), mean age was 42 ± 17 years and most patients were male (80%). The mean GCS was 7 ± 4 and the majority of patients had preserved pupillary reactivity (84%). Twenty-five percent were treated with decompressive craniectomy. The sub-cohort of 239 patients with available outcome data showed a similar demographic, admission, and treatment profile compared with the group without outcome data. In the outcome cohort, 52% had recovered favourably and 24% were deceased six months post-injury.

**Table 1:** Demography, admission variables, cerebral physiology, treatments, and outcome

Variables	Entire cohort	Outcome cohort
Patients, n (%)	425 (100%)	239 (56%)
Demography		
Age (years), mean ± SD	42 ± 17	41 ± 17
Sex (male/female), n (%)	260/65 (80/20%)	176/45 (80/20%)
Admission variables		
GCS (scale), mean ± SD	7 ± 4	7 ± 3
Pupillary reactivity (normal/1 or 2 unreactive), n (%)	150/28 (84/16%)	135/23 (85/15%)
Cerebral physiology		
Length of ICP-monitoring (days), mean ± SD	5 ± 4	5 ± 4
Length of pbtO_2_-monitoring (days), mean ± SD	5 ± 4	6 ± 4
ICP (mmHg), mean ± SD	12 ± 7	15 ± 7
PRx (coefficient), mean ± SD	0.05 ± 0.20	0.03 ± 0.21
ORx (coefficient), mean ± SD	0.22 ± 0.17	0.24 ± 0.20
CPP (mmHg), mean ± SD	76 ± 7	77 ± 7
CPPopt (mmHg), mean ± SD	76 ± 6	77 ± 6
PbtO_2_ (mmHg), mean ± SD	28 ± 12	29 ± 13
Treatments		
Decompressive craniectomy, n (%)	73 (25%)	57 (27%)
Outcome		
Favourable/unfavourable outcome, n (%)	N/A	124/115 (52/48%)
Mortality, n (%)	N/A	58 (24%)

Missing data in the entire cohort: Age (n = 62), sex (n = 100), GCS (n = 139), pupillary reactivity (n = 247), decompressive craniectomy (n = 130), GOS (n = 186)

The sub-cohort with available outcome data exhibited higher ICP (*p* < 0.001), PRx (*p* = 0.03), ORx (*p* = 0.02), CPPopt (*p* = 0.01), and pbtO_2_ (*p* = 0.02) than the sub-cohort without such data. There was no difference in age, sex, GCS, pupillary reactivity, the length of ICP- or pbtO_2_-monitoring, CPP, or the rate of decompressive craniectomy between those groups

Missing data in the outcome cohort: Age (n = 3), sex (n = 18), GCS (n = 18), pupillary reactivity (n = 81), decompressive craniectomy (n = 30)

CPP = Cerebral perfusion pressure. CPPopt = Optimal CPP. GCS = Glasgow Coma Scale. GMT = Good monitoring time. ICP = Intracranial pressure. N/A = Not applicable. ORx = Oxygen reactivity index. PbtO_2_ = Partial brain tissue oxygen. PRx = Pressure reactivity index. SD = Standard deviation

### Cerebral physiology – descriptive data

In the entire cohort (Table 1), mean ICP was 12 ± 7 mmHg, mean PRx was 0.05 ± 0.20, mean ORx was 0.22 ± 0.17, mean CPP was 76 ± 7 mmHg, mean CPPopt was 76 ± 6 mmHg, and mean pbtO_2_ was 28 ± 12 mmHg. The sub-cohort with available outcome data exhibited slightly higher ICP, PRx, ORx, CPPopt, and pbtO_2_ as compared with the group without outcome data.

### ORx in relation to cerebral physiology

**Fig. 1:**
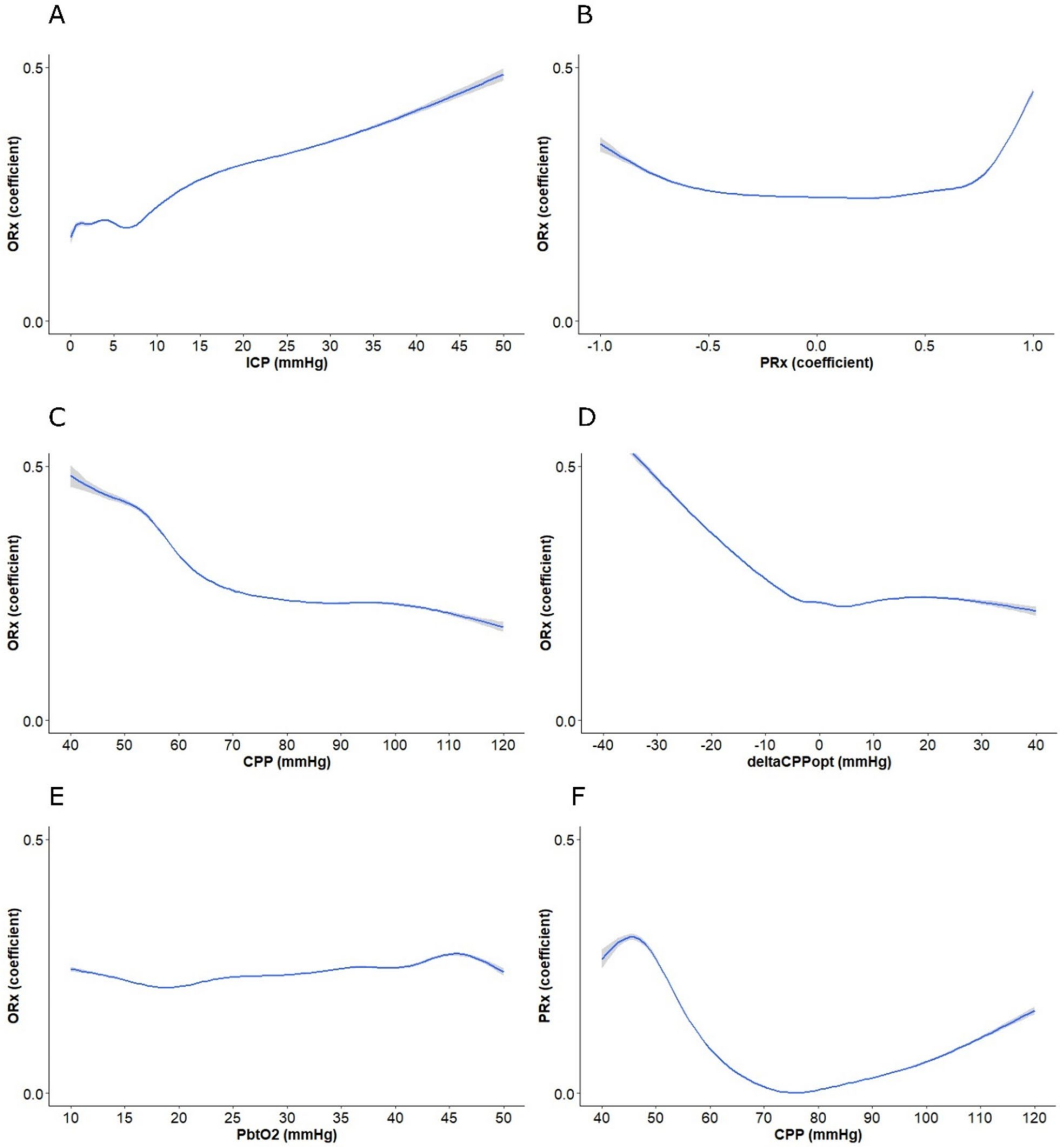
ORx in relation to cerebral physiology– GAM analyses. In these GAMs, ORx increased gradually with higher ICP (**A**), while it remained stable over the PRx range − 1.0 to + 0.5 and increased for higher PRx values. Furthermore, ORx increased in a pronounced way when CPP (**C**) went below 60–70 mmHg and when ΔCPPopt (**D**) became negative. There was only a small variation in ORx over the pbtO_2_ range (**E**), but ORx tended to increase when pbtO_2_ was below 10–15 mmHg. Lastly, a GAM of CPP vs PRx (**F**) was conducted to illustrate that while ORx only increased for low CPPs, PRx changed with CPP in a U-shaped way. CPP = Cerebral perfusion pressure. CPPopt = Optimal CPP. GAM = Generalized additive model. ICP = Intracranial pressure. PbtO_2_ = Partial brain tissue oxygen. PRx = Pressure reactivity index. ORx = Oxygen reactivity index

In the GAM analyses (Fig. 1), ORx increased gradually with higher ICP (1A), while it increased more steeply when CPP (1C) went below 60 to 70 mmHg and this was particularly pronounced when CPP fell below the PRx-based CPPopt (1D). ORx also increased slightly when ΔCPPopt was above zero, but decreased for more extreme, positive ΔCPPopt values. ORx remained relatively stable over the PRx range, but increased from PRx above + 0.30 and even more sharply for PRx >  + 0.70 (1B). ORx appeared stable throughout the pbtO_2_ range (1E). In contrast to the L-shaped association between ORx and CPP (1C), PRx, as expected, changed in a U-shaped way with CPP (1F), i.e., deteriorated both for low and high CPP values.

Linear mixed effect models with ORx as the dependent variable were conducted (Table 2). ICP, CPP, PRx above/below + 0.30, and the interaction of PR above/below + 0.30 with ICP and CPP were included as fixed effects. Patients, time from monitoring, and the specific monitoring sequence were included as random effects. After backwards stepwise reduction of non-significant fixed effects, higher ICP, lower CPP, and impaired CA (PRx >  + 0.30) were significantly associated with higher ORx. The overall explanatory value of the fixed effects was low as the marginal R^2^ was 0.01, but the conditional R^2^ was higher at 0.47, indicating that the random effects had a greater explanatory value for the ORx variations. In a similar model based on CPPopt rather than CPP, CPP both below and above CPPopt together with higher ICP were associated with higher ORx. The marginal and conditional R^2^ showed similar values as in the previous model.

**Table 2:** ORx in relation to PRx – a linear mixed effect models analysis

*Variable*	*β*	*SE*	*p*
ORx—CPP model			
Intercept	0.64	0.03	< 0.001
ICP (mmHg)	0.0025	0.0005	< 0.001
CPP (mmHg)	-0.0051	0.0003	< 0.001
PRx (intact)*	-0.0155	0.0071	0.03
ORx—CPPopt model			
Intercept	0.19	0.01	< 0.001
ICP (mmHg)	0.0029	0.0006	< 0.001
Negative ∆CPPopt (mmHg)	0.0083	0.0005	< 0.001
Positive ∆CPPopt (mmHg)	0.0020	0.0005	< 0.001

^*^PRx intact was defined as below + 0.30

“Negative ∆CPPopt (mmHg)” is described as a positive/absolute value

CPP: AIC was 65,335, conditional R^2^ was 0.47 and marginal R^2^ was 0.01

CPPopt: AIC was 57,886, conditional R^2^ was 0.47 and marginal R^2^ was 0.005

Two separate models were used to predict hourly values of ORx based on the global perfusion-related variables, one with absolute CPP and another with ∆CPPopt (mean hourly positive ∆CPPopt > 0 and negative ∆CPPopt < 0). The model based on absolute CPP-values also included ICP, PRx (above/below 0.30), and the interaction between ICP and CPP with PRx as fixed effects. Random effects were time from monitoring (slope) and patients and sequence (nested intercepts). Stepwise backward model reduction was done to exclude fixed effects variables that were not significant (*p* > 0.05) and the table shows the final model after these procedures. The model assumptions were checked with standardized residuals vs fitted values plots and with qq-plots

AIC = Akaike information criterion. CPP = Cerebral perfusion pressure. CPPopt = Optimal CPP. ICP = Intracranial pressure. ORx = Oxygen reactivity index. PRx = Pressure reactivity index. SE = Standard error

### ORx in relation to outcome

**Fig. 2:**
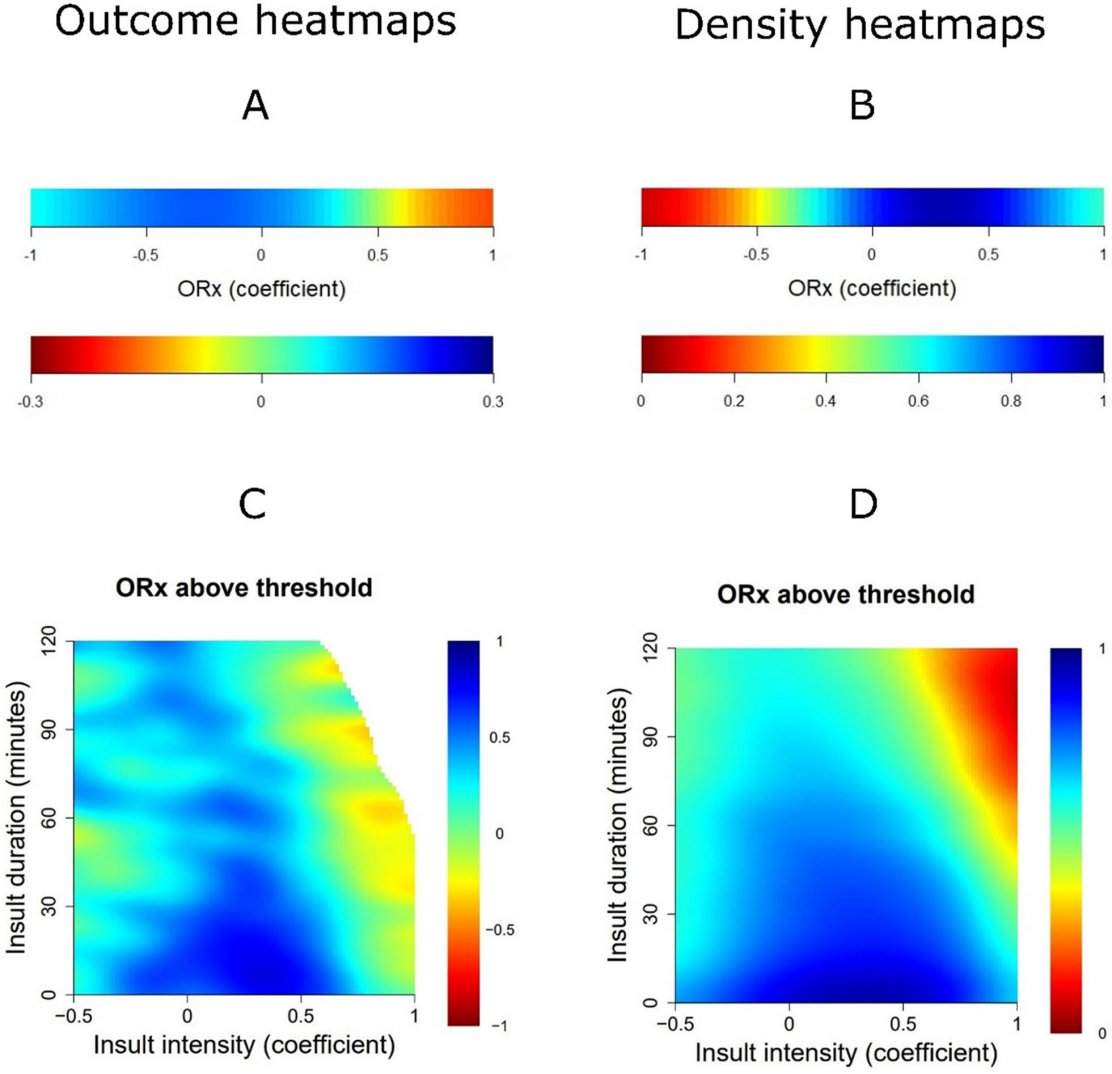
**A**–**D**. ORx in relation to outcome—GMT and insult intensity/duration analyses. ***Outcome heatmaps***—Figure **A**, this outcome heatmap illustrates that ORx above + 0.5 was particularly associated with unfavourable outcome (red colour), while lower values were more favourable (blue colour). Figure **C**, this outcome heatmap illustrates a transition towards worse outcome for ORx above approximately + 0.5 for longer durations (90 min), while a transition occurred after only a few minutes when ORx was above + 0.75. The colour code for the correlation coefficients is displayed below the outcome heatmap in Figure **A** and on the right side in Figure **C**. ***Density heatmaps***—Figure **B**, this is the corresponding density heatmap for Figure **A**, which shows that ORx was centred around − 0.25 to + 1.0. Figure **D**, this is the corresponding density heatmap to Figure **C**, which shows an overall wide data distribution, although ORx above + 0.50 for longer durations were relatively rare. Blue colour indicates the data were frequent for that variable combination, while red colour indicates the opposite. The colour code for the data density is displayed below the density heatmap in Figure **B** and on the right side in Figure **D**. GMT = Good monitoring time. ORx = Oxygen reactivity index

**Fig. 3:**
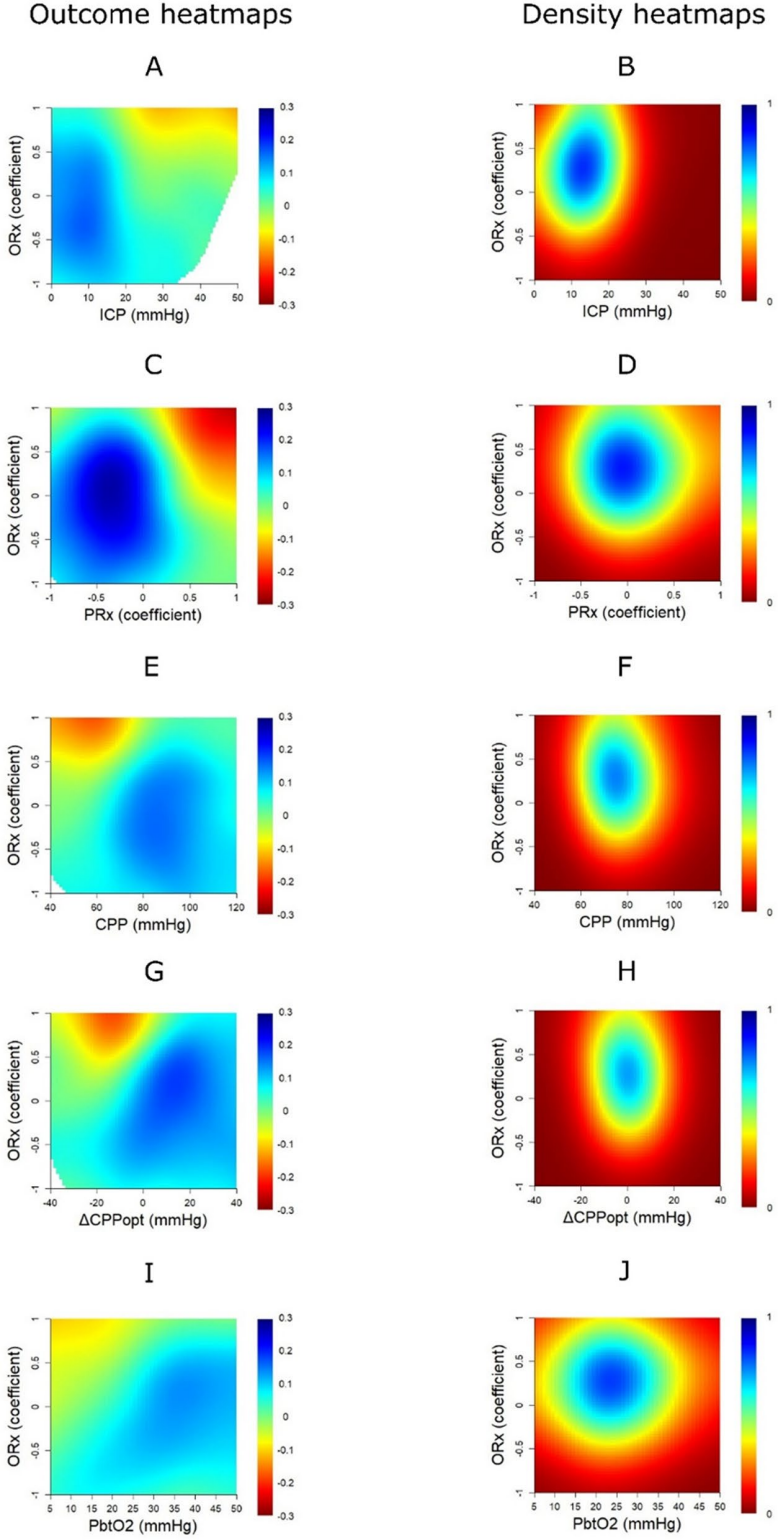
**A**–**J** The interaction between ORx and ICP, PRx, CPP, ΔCPPopt, and pbtO_2_ in relation to outcome. ***Outcome heatmaps***—These heatmaps indicate that a higher %GMT of elevated ORx combined with high ICP (**A**), high PRx (**C**), low CPP (**E**), negative ΔCPPopt (**G**), and low pbtO_2_ (**I**) were associated with worse outcome. Blue colour indicates an association between higher %GMT and higher GOS/favourable outcome, while red colour indicates the opposite association. The colour code for the correlation coefficients is displayed on the right side in the outcome heatmaps. ***Density heatmaps***—These heatmaps of ORx combined with ICP (**B**), PRx (**D**), CPP (**F**), ΔCPPopt (**H**), and pbtO_2_ (**J**) shows the data distribution. Blue colour indicates the data were frequent for that variable combination, while red colour indicates the opposite association. The colour code for the data density is displayed on the right side to the density heatmaps. CPP = Cerebral perfusion pressure. CPPopt = Optimal CPP. ΔCPPopt = CPP-CPPopt. GOS = Glasgow outcome scale. ICP = Intracranial pressure. PbtO_2_ = Partial brain tissue oxygen. PRx = Pressure reactivity index. ORx = Oxygen reactivity index

In the outcome heatmaps, a higher %GMT of ORx above + 0.50 was associated with lower GOS (Fig. 2A). Similarly, there was a transition towards worse outcome when ORx exceeded + 0.50 for episodes of 90 min, while this transition occurred at lower durations for higher intensities (Fig. 2C). Figure 2B and D illustrate the corresponding data distribution for these outcome heatmaps. Furthermore, higher ORx in combination with ICP above 20 mmHg (Fig. 3A), PRx above + 0.30 (Fig. 3C), CPP below 60–70 mmHg (Fig. 3E), negative ΔCPPopt (Fig. 3G), and pbtO_2_ below 20 mmHg, though weakly in this case, (Fig. 3I) were particularly associated with worse outcome in the two-variable heatmaps. The corresponding density heatmaps for ORx in combination with ICP (Fig. 3B), PRx (Fig. 3D), CPP (Fig. 3F), ΔCPPopt (Fig. 3H), and pbtO_2_ (Fig. 3J) are demonstrated in the same figure.

In a multivariable logistic outcome regression (Table 3), mean ORx (OR [95% CI] = 11.3 [1.82–73.67], *p* = 0.01) was independently associated with mortality, after adjustment for age, GCS, and PRx. However, ORx was not independently associated with favourable outcome.

**Table 3:** ORx in relation to favourable outcome and mortality –multivariable logistic regression analyses

Variables	Favourable outcome		Mortality	
	OR (95% CI)	*p*	OR (95% CI)	*p*
Age (years)	0.97 (0.96–0.99)	***0.003***	1.02 (0.999–1.04)	0.07
GCS (scale)	1.15 (1.06–1.26)	***0.002***	0.92 (0.82–1.03)	0.15
PRx (coefficient)	0.06 (0.01–0.31)	***0.001***	161.51 (25.86–1271.11)	** < *0.001***
ORx (coefficient)	0.41 (0.09–1.84)	0.25	11.31 (1.82–73.67)	***0.01***

Favourable outcome; AUROC (95% CI) = 0.73 (0.67–0.80), AIC = 271, Nagelkerke = 0.35

Mortality (AUROC (95% CI) = 0.80 (0.73–0.88), AIC = 185, Nagelkerke = 0.35

AIC = Akaike information criterion. AUROC = Area under receiver operating characteristics curve. CI = Confidence interval. GCS = Glasgow Coma Scale. OR = Odds ratio. ORx = Oxygen reactivity index. PRx = Pressure reactivity index

## Discussion

In this large TBI study, ORx increased with higher ICP, higher PRx, lower CPP, and negative ΔCPPopt, but only to a smaller extent for positive ΔCPPopt and not for higher CPP. These findings indicate that ORx is primarily sensitive to the lower, but not the upper, limit of autoregulation, in contrast to PRx which was sensitive to both. The combination of high ORx with elevated ICP, high PRx, low CPP, negative ΔCPPopt, and low pbtO_2_ was particularly associated with worse outcome than any of these physiological disturbances alone. Thus, ORx may be useful to determine the safe and dangerous perfusion target intervals and could have a complementary value to the global CA metric PRx.

### ORx in relation to cerebral physiology

The GAMs revealed that ORx deteriorated with higher ICP, CPP below 60–70 mmHg, and negative ΔCPPopt, which also held true in the linear mixed effect models. These findings corroborate that ORx is sensitive to the lower limit of CA, which was anticipated, since CPP is expected to change in the same direction as pbtO_2_ in that scenario. However, ORx remained stable and low when CPP was above 60 mmHg, i.e., above the estimated lower limit of CA on group level [34]. ORx increased minimally for slightly positive ΔCPPopt, but thereafter decreased for more extreme ΔCPPopt-values, while there was a positive association between positive ΔCPPopt and ORx in the linear mixed effect model. This discrepancy can be explained by the limitation of linear assumption in the latter model. When the upper limit of CA is exceeded, higher CPP may increase CBF and pbtO_2_ (leading to higher ORx) to some extent. However, pbtO_2_ may rapidly become stable and saturated as the vascular and tissue oxygen gradient gradually decreases [22], which would result in relatively low ORx. Consistent with this idea, ORx, overall, remained low and only slight changes could be seen for very high CPP and positive ΔCPPopt. Altogether, ORx appeared to be sensitive to the lower, but not the upper, limit of CA and displayed an L-shaped association with CPP. In contrast, and as expected [12, 13], PRx showed a U-shaped association with CPP. This finding suggests that PRx is sensitive to both the lower and upper limit of reactivity, which may partly explain why ORx and PRx were only moderately associated with each other. Another important factor that could explain the limited overlap between ORx and PRx is that pbtO_2_ and thereby ORx not only depends on CPP and CBF, but also arterial oxygen content and focal energy metabolism. Thus, the effect of systemic oxygenation from arterial blood gases and focal energy metabolism with cerebral microdialysis in relation to ORx need to be explored in future studies.

### ORx in relation to outcome

There was a clear transition towards worse outcome when ORx exceeded + 0.50, particularly for longer episodes. Furthermore, clinical outcome was worse for patients who exhibited combined insults with higher %GMT of elevated ORx together with ICP above 20 mmHg, PRx above + 0.30, CPP below 60–70 mmHg, negative ΔCPPopt, and pbtO_2_ below 20 mmHg. All of these findings suggest that ORx carries important prognostic information, most likely indicating a greater burden of ischaemic brain injury as the lower limit of CA is exceeded. It also means that ORx could potentially be used to aid in fine-tuning the safe and dangerous perfusion pressure and pbtO_2_ targets. Of particular interest, the combined ORx/PRx plot demonstrated an additive or synergistic effect when both of these variables were elevated at the same time as compared to isolated insults. One reason for this effect could be that PRx carries a low signal-to-noise ratio [35]. Adding ORx, as a complementary variable of CA, based on different input variables, could help in differentiating between the true physiological signal as compared to noise for PRx. In addition, ORx is based on the continuous correlation of 30-s values between CPP and pbtO_2_ over 1 h, as opposed to 10 s-values of ABP and ICP over 5 min. Arguably, ORx rather reflects slow-changes to CPP and “static” CA, while PRx may be more indicative of “dynamic” CA. Furthermore, PRx is a volume-based metric of vascular reactivity, while ORx is rather a CBF- and oxygen diffusion-based index. This is relevant, as a recent experimental pig study [36] showed that CBF starts to deteriorate slightly before the entire vasodilatory reserve is exhausted when CA is still only slightly impaired. Thus, cerebral ischaemia and hypoxia could occur for low CPP when PRx is still relatively low and ORx could potentially add information in this scenario. Lastly, ORx is based on focal pbtO_2_-monitoring and therefore primarily valid for a small, restricted brain area, whereas PRx is a global CA variable. Focal monitoring methods may not always be sensitive to distant or global pathophysiological events, while global methods may not be sensitive to such disturbances in small, but important brain areas. Thus, as indicated in our results and in previous studies, there seems to be an added value when global and focal monitoring variables are analysed together [20, 27, 37]. Particularly, pbtO_2_-derived data, including ORx, appears mostly relevant to fine-tune management when the entire brain is affected by concurrent global physiological disturbances.

Furthermore, the role of PRx and ORx in aSAH have recently been explored [9, 15, 38]. Of particular interest and in contrast to TBI, PRx seems to demonstrate only weak associations with important clinical outcomes in aSAH [15, 38, 39]. It has been suggested that the ABP/ICP correlation may be less reliable in case of lower ICP values with reduced variability, which may more often be the case in aSAH than in TBI [15]. Another explanation is that while PRx is a global CA metric, it might not be sufficiently sensitive to detect focal ischaemia which may be common in aSAH [38]. On the contrary, ORx has recently proven to be useful to predict the risk of developing delayed cerebral ischaemia and poor outcome in aSAH [15, 38, 39]. Since aSAH patients typically exhibit a right-shifted autoregulatory curve and require higher CPP to stay above the lower limit of CA [1, 9], it appears plausible that ORx as an indicator of the lower limit of CA may be particularly suitable in such a disease. However, TBI patients often exhibit CA impairment with both ischaemic and hyperaemic insults [4] and PRx is probably superior in such a scenario, although there may still be a complementary value with ORx. This idea was consistent with the results from our multiple logistic regressions, in which PRx turned out as a significant variable that was independently associated with both mortality and favourable outcome, while ORx only added significant complementary value in the mortality but not the favourable outcome analysis.

### New insights and study strengths

Initially, Jaeger et al. demonstrated in a smaller study that ORx was a promising new metric of CA that was strongly related to PRx and patient outcome in TBI [14]. However, in the subsequent follow-up studies, their findings could not be replicated and the interest in this variable decreased [16–18]. Notably, all of the previous studies were based on cohorts with less than 100 patients and focused on the effects of ORx as a single-variable [14, 16–18]. In our study 425 patients were included in the physiological analyses and 239 in the outcome analyses, reducing the risk of type I and II errors. We also used advanced statistical models to investigate associations between ORx and multimodal cerebral physiology, taking into account potentially non-linear relations (GAMs) and random effects (patients and time; linear mixed effect models), making our results more detailed and robust. Furthermore, granular visualization techniques were used to demonstrate the complex association between ORx and outcome over the entire monitoring, for specific threshold intensities/durations, and in combination with other cerebral physiological variables. Thus, with our large cohort and new analytical approaches, it appears clearer that ORx adds clinically relevant information in moderate to severe TBI. Particularly, ORx seems to be sensitive to the lower limit of autoregulation and to indicate the risk of imminent cerebral ischaemia, when analysed in combination with other global cerebral physiological variables.

### Limitations

Due to ethical restrictions, we were unable to retrieve variables on demography, admission status, imaging features, and treatments in some cases. Particularly, it would have been interesting to differentiate ORx values based on the proximity of the probe to focal lesions, as this factor may influence the pbtO_2_ and ORx values [40]. There have also been some concerns whether different monitors (e.g., Licox vs. Raumedic) yield different ORx values [41], possibly due to differences in reaction time to CBF changes in vivo. Thus, the external validity of our results may be limited to ORx monitoring with the Licox probe. Furthermore, although the reliability of PRx and CPPopt post-craniectomy has been questioned, a previous study suggests that it is preserved [42] and we therefore decided to keep the craniectomy patients in the analyses. In addition, GAMs may become unreliable in areas with low-density data and they should therefore be interpreted cautiously in areas with wider CI. Similarly, low-density areas should also be interpreted with caution in the outcome heatmaps (Figs. 2 and 3). However, we did set thresholds for minimum number of patients with insults to mitigate this issue, at least partially. Furthermore, there is usually a plethora of factors responsible for outcome prognosis and the fact that the correlation coefficient between cerebral physiological variables and outcome was modest in the outcome heatmaps was expected. In addition, only half of the patients had available outcome data. This subgroup did not differ in demography, admission status, or treatments in comparison to the cohort with missing outcome data, but the former group exhibited slightly worse cerebral physiology. Lastly, the study spanned over 20 years and the general management changed to some extent during this period. Particularly, the ABP reference point was altered from atrial to tragus level. This implies that the reliability of the analyses related to CPP, but not ICP, PRx, ΔCPPopt, and pbtO_2_, was affected to some extent.

## Conclusions

ORx was sensitive to the lower, but not the upper, limit of CA, in contrast to PRx which was sensitive to both. The combination of high ORx with elevated ICP, high PRx, low CPP, negative ΔCPPopt, and low pbtO_2_ was particularly associated with worse outcome. Thus, pbtO_2_-monitoring with the corresponding ORx metric may be useful during neurocritical care in TBI, to determine the safe and dangerous perfusion target intervals and it may add a complementary value to the global cerebral pressure autoregulatory metric PRx.

## Supplementary Information

Additional file 1 (PDF 48 KB)

## Data Availability

Data are available upon reasonable request.

## Abbreviations

ABP: Arterial blood pressure
AIC: Akaike information criterion
aSAH: Aneurysmal subarachnoid haemorrhage
CA: Cerebral autoregulation
CBF: Cerebral blood flow
CI: Confidence interval
CPP: Cerebral perfusion pressure
CPPopt: Optimal CPP
GAM: Generalized additive model
GCS: Glasgow Coma Scale
GMT: Good monitoring time
GOS: Glasgow Outcome Scale
ICP: Intracranial pressure
IQR: Interquartile range
NCC: Neurocritical care
OR: Odds ratio
ORx: Oxygen reactivity index
PbtO_2_: Partial brain tissue oxygenation
PRx: Pressure reactivity index
SD: Standard deviation
SE: Standard error
TBI: Traumatic brain injury
