# Correction: Revisiting the oxygen reactivity index in traumatic brain injury: the complementary value of combined focal and global autoregulation monitoring

**DOI:** 10.1186/s13054-025-05614-1

**Published:** 2025-08-21

**Authors:** Teodor Svedung Wettervik, Erta Beqiri, Anders Hånell, Stefan Yu Bögli, Ihsane Olakorede, Xuhang Chen, Adel Helmy, Andrea Lavinio, Peter J. Hutchinson, Peter Smielewski

**Affiliations:** 1 Department of Medical Sciences, Section of Neurosurgery, Uppsala University, 751 85 Uppsala, Sweden; 2 Brain Physics Laboratory, Department of Clinical Neurosciences, Division of Neurosurgery, University of Cambridge, Cambridge, UK; 3 Department of Clinical Neurosciences, Division of Neurosurgery, Addenbrooke’s Hospital, University of Cambridge, Cambridge, UK; 4 Neurosciences and Trauma Critical Care Unit, Addenbrooke’s Hospital, Cambridge University Hospitals, Cambridge, UK

**Correction: Critical Care (2025) 29:20**

10.1186/s13054-025-05261-6

Following publication of the original article [1], the authors noticed that the 95% confidence interval of ORx was slightly wrong in Table 3, which was revised in the published article. However, the authors realized that they did not update this reference in the main text of the **Results** > **ORx in relation to outcome** section.

The last paragraph in the **Results** > **ORx in relation to outcome** section currently reads:

In a multivariable logistic outcome regression (Table 3), mean ORx (OR [95% CI] = 11.3 [25.87–12,271.11], *p* = 0.01) was independently associated with mortality, after adjustment for age, GCS, and PRx. However, ORx was not independently associated with favourable outcome.

The last paragraph in the **Results** > **ORx in relation to outcome** section should read:

In a multivariable logistic outcome regression (Table 3), mean ORx (OR [95% CI] = 11.3 [1.82–73.67], *p* = 0.01) was independently associated with mortality, after adjustment for age, GCS, and PRx. However, ORx was not independently associated with favourable outcome.

The last paragraph in the Results > ORx in relation to outcome section has been updated in this correction article and the original article [1] has been corrected.
