# Supplementary material for: Revisiting the oxygen reactivity index in traumatic brain injury: the complementary value of combined focal and global autoregulation monitoring
Source: Crit Care. 2025 Jan 12;29:20. doi: 10.1186/s13054-025-05261-6 (PMC11725216; doi:10.1186/s13054-025-05261-6)
Supplement: Supplementary file 1 — Additional file 1 (PDF 48 KB) [file 13054_2025_5261_MOESM1_ESM.pdf]

Additional file 1. Flowchart of patient inclusion

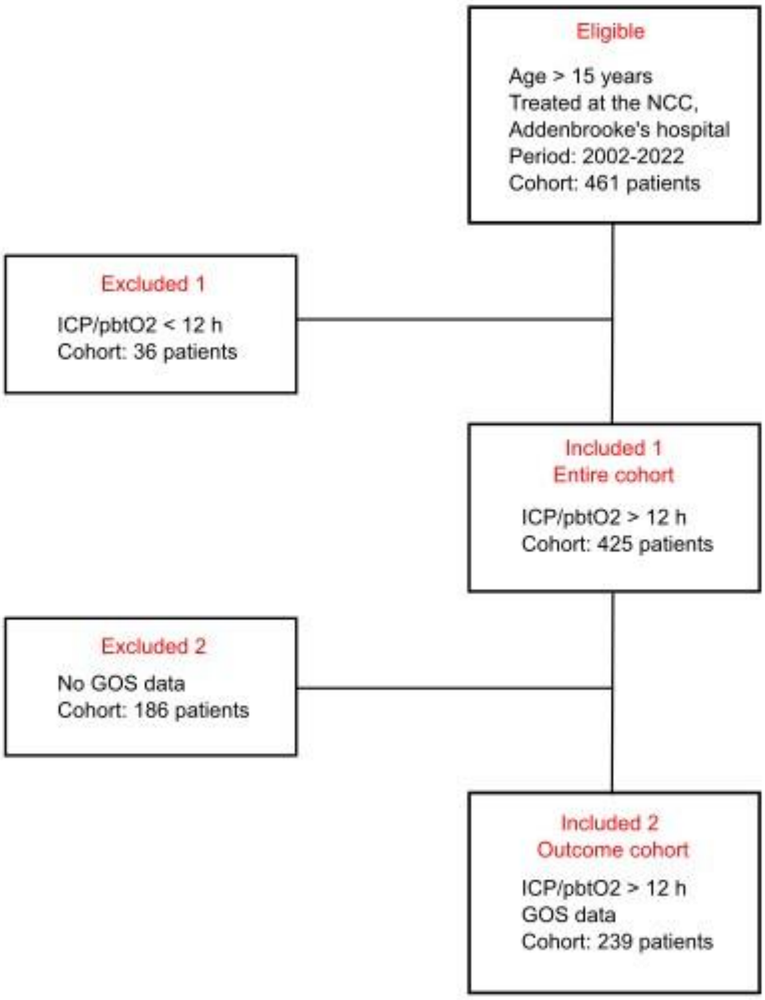

GOS = Glasgow Outcome Scale. ICP = Intracranial pressure. NCC = Neurocritical care. $PbtO_2$ = Partial brain tissue oxygenation.
